# Wicked: The untold story of ciprofloxacin

**DOI:** 10.1371/journal.ppat.1006805

**Published:** 2018-03-01

**Authors:** Zachary C. Conley, Truston J. Bodine, Andrew Chou, Lynn Zechiedrich

**Affiliations:** 1 Verna and Marrs McLean Department of Biochemistry and Molecular Biology, Baylor College of Medicine, Houston, Texas, United States of America; 2 Department of Molecular Virology and Microbiology, Baylor College of Medicine, Houston, Texas, United States of America; 3 Interdepartmental Program in Translational Biology and Molecular Medicine, Baylor College of Medicine, Houston, Texas, United States of America; 4 Medical Scientist Training Program, Baylor College of Medicine, Houston, Texas, United States of America; 5 Michael E. DeBakey Veterans Affairs Medical Center, Houston, Texas, United States of America; 6 Department of Pharmacology and Chemical Biology, Baylor College of Medicine, Houston, Texas, United States of America; The University of North Carolina at Chapel Hill, UNITED STATES

## No one solves the wicked

“Are [bacteria] born wicked? Or do they have wickedness thrust upon them?”

–*Wicked*

Antibiotics, long used in an attempt to solve the problem of bacterial infections, have resulted in the unintended consequence of a worldwide spread of antibiotic resistance. The problem of antibiotic resistance, like many societal problems, cannot be solved; it can be only improved or worsened. This problem is now so dire that it meets the definition of a “super-wicked” problem [1]. Whereas problems that are classified as “wicked” have several defining characteristics (Table 1), super-wicked problems are additionally notable because time is running out, no one is in charge, those trying to solve the problems are causing them, and approaches have unknowable future consequences.

**Table 1:** Characteristics of a wicked problem.

1	**There is no definitive formulation of a wicked problem.** It is not possible to write a well-defined statement of the problem, as can be done with an ordinary problem.
2	**Wicked problems have no stopping rule.** You can tell when you have reached a solution with an ordinary problem. With a wicked problem, the search for solutions never stops.
3	**Solutions to wicked problems are not true or false, but good or bad.** Ordinary problems have solutions that can be objectively evaluated as right or wrong. Choosing a solution to a wicked problem is largely a matter of judgment.
4	**There is no immediate and no ultimate test of a solution to a wicked problem.** It is possible to determine right away if a solution to an ordinary problem is working. But solutions to wicked problems generate unexpected consequences over time, making it difficult to measure their effectiveness.
5	**Every solution to a wicked problem is a “one-shot” operation; because there is no opportunity to learn by trial and error, every attempt counts significantly.** Solutions to ordinary problems can be easily tried and abandoned. With wicked problems, every implemented solution has consequences that cannot be undone.
6	**Wicked problems do not have an exhaustively describable set of potential solutions, nor is there a well-described set of permissible operations that may be incorporated into the plan.** Ordinary problems come with a limited set of potential solutions, by contrast.
7	**Every wicked problem is essentially unique.** An ordinary problem belongs to a class of similar problems that are all solved in the same way. A wicked problem is substantially without precedent; experience does not help you address it.
8	**Every wicked problem can be considered to be a symptom of another problem.** While an ordinary problem is self-contained, a wicked problem is entwined with other problems. However, those problems do not have one root cause.
9	**The existence of a discrepancy representing a wicked problem can be explained in numerous ways.** A wicked problem involves many stakeholders, who all will have different ideas about what the problem really is and what its causes are.
10	**The planner has no right to be wrong.** Problem-solvers dealing with a wicked issue are held liable for the consequences of any actions they take because those actions will have such a large impact and are hard to justify.

Of the three general strategies used to cope with wicked problems (Table 2), the collaborative method, in which all stakeholders must come together, is often the last attempted. The reason for this delay is explained, in part, because it is the only method that does not turn the challenge of solving the problem over to a select few authorities but is mostly explained by the fact that it is very difficult to do. Regarding the fight against antibiotic resistance, the authoritative and the competitive strategies have been attempted. Unfortunately, the world has now “failed into the collaborative strategy” [2]. Although complex and time-consuming, a collaborative approach has no room for individual interests, demands a “community of interest,” and requires a willingness to work together with integrity, humility, and courage [2].

**Table 2:** Strategies for coping with wicked problems.

**Authoritative**	Puts the problem in the hands of a few. This approach reduces the complexity of the problem-solving, thus saving time, but authorities can be wrong with devastating consequences.
**Competitive**	This zero-sum game, or win–lose strategy, means that power, however achieved, controls the problem-solving. Competitions among power-seekers consume time and resources that could be applied to solving the problem.
**Collaborative**	A win–win collaborative approach allows cost and benefit sharing but takes longer and relies on excellent communication skills.

We write this review to engage not only our fellow researchers and clinicians but also the public and officials so that we can all come together to improve the situation. Much of the work, including our own, toward understanding fluoroquinolone resistance has been done with *Escherichia coli* (one of the most frequent sources of infection), so most of the data below are from this important microbe.

## What is this treatment?

Ciprofloxacin (international brand names: Cipro, Ciprobay, Ciproxin, Ciproxan, Bactiflox, etc.) is a broad-spectrum antibiotic of the semisynthetic fluoroquinolone class. It is one of the most widely used antibiotics in the world because of its efficacy, safety, and relatively low cost. Yet concurrent with its widespread use, resistance to this excellent drug has rendered it less effective, causing worry for its further utility. To reduce drug resistance, healthcare providers and patients alike must become good stewards of antibiotics, which requires an increased understanding of how they work and the unintended personal and societal consequences of their use.

## The topoisomerase and I

Fluoroquinolones, like ciprofloxacin, have two bacterial drug targets, DNA gyrase and DNA topoisomerase IV [3,4], both of which are essential enzymes. Although most sequenced bacteria have both enzymes, some, such as mycobacteria that cause tuberculosis, have only DNA gyrase. By passing DNA strands through each other, DNA topoisomerases keep DNA untangled and modulate torsional stress to affect DNA replication, transcription, and, plausibly, every other aspect of DNA metabolism (reviewed in [5,6]). Passing DNA strands through each other involves the enzyme making double-strand DNA breaks, which is dangerous because these breaks can be highly genotoxic. Topoisomerases usually mitigate this risk by maintaining the breaks only transiently. Fluoroquinolones interfere with the transient nature of the topoisomerase-induced double-strand breaks by stabilizing a DNA/topoisomerase/drug ternary complex that traps the enzyme-bound double-strand breaks, thereby blocking bacterial growth (a "bacteriostatic" effect) (reviewed by [7,8]). Fluoroquinolone treatment also increases reactive oxygen species, resulting in bacterial death (a “bactericidal” effect) (reviewed in [9]). The understanding of the relative contributions of stabilized DNA breaks or increased reactive oxygen species to successful fluoroquinolone treatment is incomplete.

## Something bad

When ciprofloxacin fails to cure an infection, healthy individuals typically can take a different antibiotic to treat the infection without lasting consequences. Patients with certain medical conditions (for example, cancer, organ transplant, autoimmune disease, diabetes mellitus, advanced age, or severe malnutrition), however, may be at increased risk for infection-related complications and/or death. Identifying a bacterial infection, deciding whether or not to use an antibiotic, and, if so, choosing which antibiotic to use are critically important tasks that have long-lasting societal impact.

When a patient has a potential infection, hospital microbiology laboratory personnel use automated systems for bacterial species determination and antibiotic susceptibility testing (AST) to help determine proper treatment. AST systems measure the antibiotic minimum inhibitory concentration (MIC), which is the concentration needed to stop visible bacterial growth, and interpret the MICs into categories used for clinical guidance—susceptible (S), intermediate (I), or resistant (R). These categories are made using criteria set forth by antimicrobial standards groups, the Clinical & Laboratory Standards Institute (CLSI), and the European Committee on Antimicrobial Susceptibility Testing (EUCAST) [10,11]. According to CLSI, the S/I/R breakpoints for ciprofloxacin in *E*. *coli*, for example, are 1 μg/mL, 2 μg/mL, and 4 μg/mL, respectively; for EUCAST, the susceptible breakpoint is ≤0.25 μg/mL, and the resistant breakpoint is >0.50 μg/mL [10,11].

The process of culturing bacteria and performing the phenotypic resistance testing usually takes two days. In rural areas, the time it takes to transport the sample to a microbiology laboratory further delays testing. Most patients are prescribed immediate treatment for their potentially serious infection before this AST is completed, a practice known as empiric prescribing. As a result, the patient may be treated initially with an antibiotic that has little to no effect because there was no bacterial infection (it is difficult to distinguish bacterial infections from viral infections), the drug chosen was not effective against that bacterium (some antibiotics work only on gram-positive or gram-negative organisms), or the infection was resistant to the empirically prescribed drug. Empiric prescribing is an important driver of antibiotic resistance and should be avoided if the infection is not critical and the patient is healthy.

**Fig 1:**
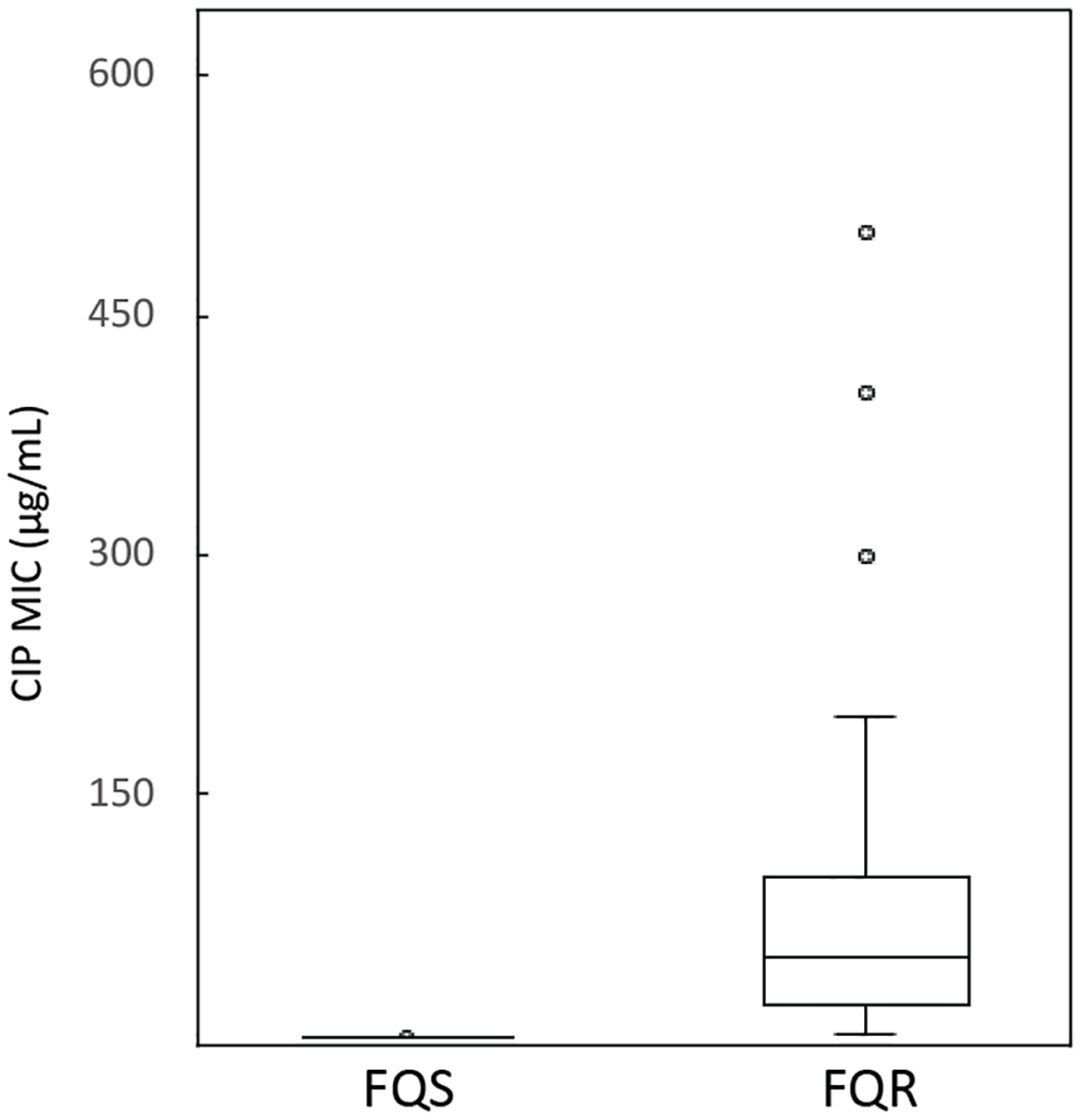
Ciprofloxacin MICs of *E*. *coli* clinical isolates. Box and whisker plots show the range of ciprofloxacin MICs for FQS and FQR clinical isolates. Data are from Becnel Boyd et al. 2009 [12]. Plots are divided into four quartiles, each representing 25% of the MICs (Q1: end of lower whisker to edge of box; Q2: edge of box to median line; Q3: median line to edge of box; Q4: edge of box to end of upper whisker). The length of the box is referred to as the IQR. Circles indicate outliers (here MICs higher than Q4 plus 1.5x IQR). Ciprofloxacin-susceptible isolates (MIC ≤1 μg/ml), as determined in the microbiology laboratory of the hospital; ciprofloxacin-nonsusceptible isolates were categorized as FQR. CIP, ciprofloxacin; FQR, fluoroquinolone-resistant; FQS, fluoroquinolone-susceptible; IQR, interquartile range; MIC, minimum inhibitory concentration.

Perhaps as a consequence of MICs being interpreted as S, I, or R, most people consider antibiotic resistance to be a false dilemma between susceptible and resistant bacteria. In actuality, ciprofloxacin MICs fall into a wide numeric continuum that do not fall neatly into these two states. For example, ciprofloxacin MICs for clinically isolated *E*. *coli* range from approximately 0.01 μg/mL to approximately 500 μg/mL and every tested concentration in between (Fig 1). Each different MIC may represent a different ciprofloxacin resistance mechanism, different combinations of resistance mechanisms, or resistance mechanisms combined with other processes that affect the resistance mechanisms or that affect the drug. No matter what the different MICs represent, the wide variation illustrates the complexity of the problem.

## I’m not that bug

There is no known single mechanism (acquired gene or allelic variation) that will change a bacterium from S to R for ciprofloxacin or any other fluoroquinolone. Instead, the genetic basis for fluoroquinolone resistance appears to be additive, with genetic alterations that alone cause modest (2- to 32-fold) increases in MIC combining with other alterations in a "step-wise" fashion [13,14].

Nearly 40 years ago, the first gene variant shown to increase fluoroquinolone MICs was discovered in *gyrA*, one of the genes encoding DNA gyrase [4]. Over a decade later, mutations in *parC*, one of the genes encoding topoisomerase IV, were discovered [3,15]. These *parC* mutations, however, do not affect fluoroquinolone MICs in the bacterium unless a mutation in *gyrA* is also present [3,16]. Fluoroquinolone resistance–associated mutations occur in specific regions of the *gyrA* and *parC* genes, known as the quinolone resistance-determining region (QRDR), and these changes may impart subtle conformational changes that affect drug and/or DNA binding [17]. Any single or pair of mutations in the QRDR, each causing up to an approximate 10-fold increase in fluoroquinolone MIC, is insufficient to reach the R breakpoints, but a combination of three or four different mutations (two in *gyrA* and one or two in *parC*) can achieve R MIC levels ([16] and references therein).

Bacterial membranes provide a barrier that protects cells from antibiotics. Membrane regulation or alteration, as well as various compounds, however, can alter the membrane to affect antibiotic permeability [18]. Porins, located in the outer membrane, selectively allow nutrients (and some antibiotics) into gram-negative bacteria. These bacteria can down-regulate, mutate, or even delete porins to decrease drug uptake (reviewed in [18,19]). Fluoroquinolones are thought to enter the bacterial cell both by crossing the membranes and by accessing the porins [18]. In *E*. *coli*, for example, the outer membrane porins OmpC and OmpF, and possibly additional porins, transport fluoroquinolones [18].

Increased drug efflux from the cell can also increase fluoroquinolone MICs. Stable or transient overexpression of the chromosomally encoded bacterial efflux pump gene, *acrAB*, in *E*. *coli* leads to increased transport of multiple different drugs, including fluoroquinolones, out of the bacterial cell [20–22], possibly as a consequence of fluoroquinolones resembling normal cell–cell communication molecules [21]. Expression of the *acrAB* genes or their homologs in other gram-negative bacteria is regulated by a complex network of genes that change in response to different environmental signals [19]. Alterations in these regulatory proteins also affect antibiotic MICs [23–26]. Increased levels of *acrAB* increase resistance to both fluoroquinolones and nonfluoroquinolone antibiotics and have been proposed to be a biomarker of multidrug resistance [27].

Ciprofloxacin resistance may also be transferred on DNA among bacteria of the same or different genus or species. This transfer is often via plasmids encoding resistance to several different antibiotics. One plasmid-encoded fluoroquinolone resistance gene is *qnr*, which encodes a protein that mimics B-form DNA. Binding of gyrase to Qnr prevents inhibition by ciprofloxacin [28]. Additional plasmid-encoded quinolone resistance mechanisms include the quinolone efflux pump A (QepA) and the olaquindox-resistant efflux pump proteins A and B (OqxAB) [29,30], as well as an aminoglycoside-modifying acetyltransferase, encoded by the *aac(6’)-Ib-cr* gene, that inactivates ciprofloxacin by acetylating it [31].

**Fig 2:**
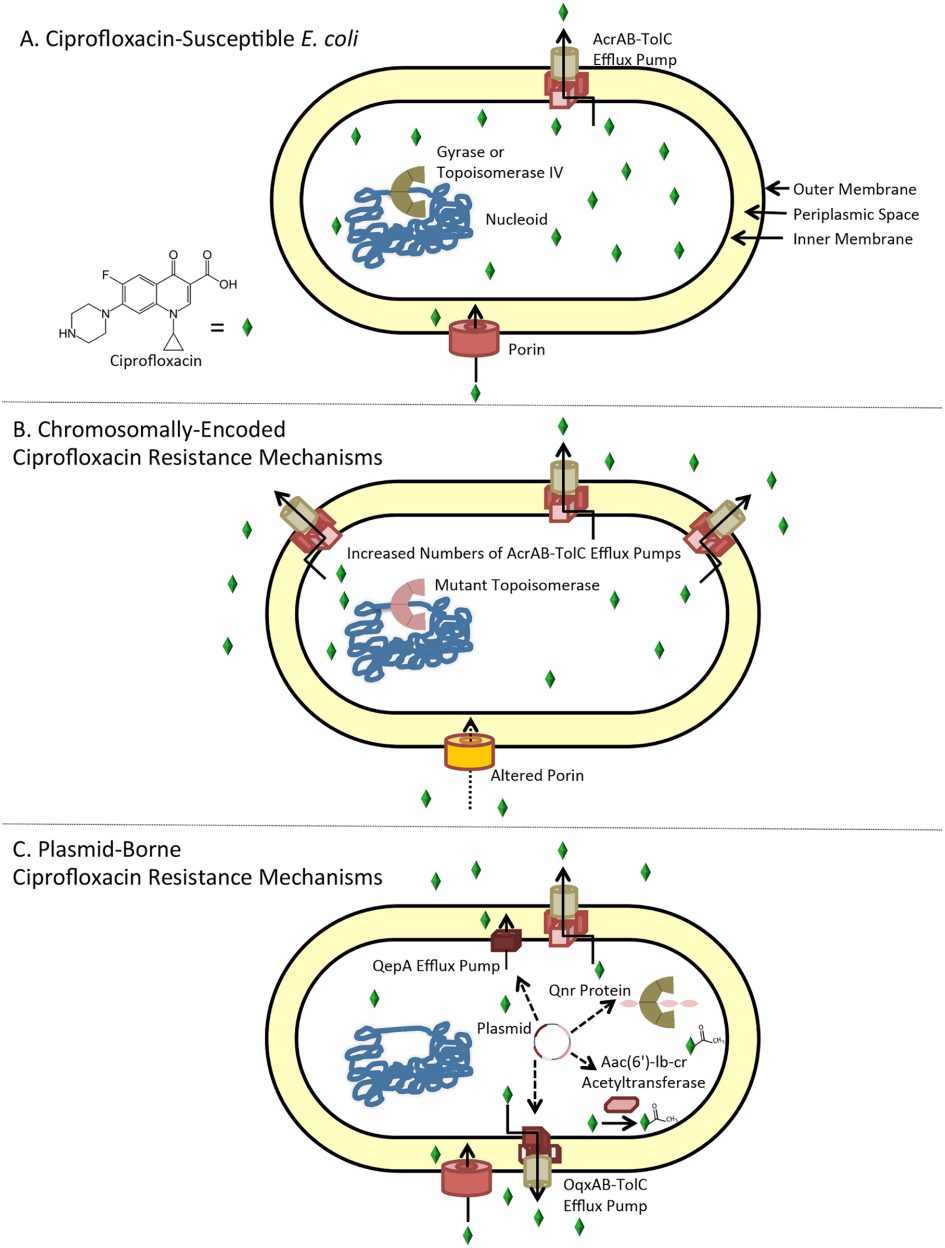
Schematic showing known ciprofloxacin resistance mechanisms in *E*. *coli*. (A) Ciprofloxacin-susceptible *E*. *coli*. The inner and outer membranes intrinsically protect the bacterium. Also depicted are the AcrAB-TolC efflux pump, porin, and DNA gyrase (or topoisomerase IV) interacting with the DNA nucleoid (in blue). Ciprofloxacin (green diamond) can diffuse through the membranes but also accesses the cell via porins. Ciprofloxacin forms a ternary complex with the topoisomerase bound to DNA, resulting in cell death. (B) Chromosomally encoded ciprofloxacin resistance mechanisms. Altered porin(s), mutant gyrase (and perhaps also topoisomerase IV), and increased numbers of AcrAB-TolC efflux pumps are shown. Ciprofloxacin access is reduced via alterations (deletion, down-regulation, or mutation) in porins. Ciprofloxacin that enters the cell can be removed through increased numbers of efflux pumps. Ciprofloxacin that reaches the mutant topoisomerase(s) is less effective against the mutant version of the enzyme than the drug-susceptible version shown in A. (C) Plasmid-borne ciprofloxacin resistance mechanisms. Plasmids can harbor genes encoding the ciprofloxacin efflux pumps QepA or OqxAB, the Qnr protein—which binds gyrase by mimicking B-form DNA—or Aac(6’)-Ib-cr, an aminoglycoside-modifying acetyltransferase that acetylates and inactivates ciprofloxacin. Aac(6’)-Ib-cr, aminoglycoside 6’-N-acetyltransferase type lb-cr; AcrAB-TolC, Acriflavin-resistant Proteins AB Tolerant to Colicin E mutant; OqxAB, olaquindox-resistant efflux pump proteins A and B; QepA, quinolone efflux pump A; Qnr, quinolone resistance protein.

The above brief introduction to the known fluoroquinolone resistance mechanisms in *E*. *coli* is summarized in Fig 2. We refer the reader to more in-depth reviews [14,18,19,32] for more detailed descriptions of the mechanisms. Other unknown mechanisms of resistance must exist, however. The known mechanisms of fluoroquinolone resistance described above only account for 50% to 70% of elevated drug MICs of *E*. *coli* clinical isolates [12,33]. Therefore, as many as half of these pathogens may contain novel ciprofloxacin resistance mechanisms. Curiously, fluoroquinolone MICs of some clinical isolates are lower than expected given the identified resistance mechanisms therein [12,33]. It would be highly advantageous to understand how cells might lower MICs or to identify any acquired genes and/or allelic variants associated with antibiotic susceptibility because these would have enormous clinical applications [33,34].

## No good deed (goes unpunished)

Even when antibiotic treatment (the first, second, or third attempt) does kill the infecting microbe, there are unintended negative consequences. Antibiotics invariably kill off some "friendly" antibiotic-susceptible bacteria, not causing infection, that are part of a healthy microbiome. Enough of this bacterial killing causes a condition of microbial imbalance known as dysbiosis, which can persist for as long as one year following an antibiotic treatment. Infection by *Clostridioides difficile* (formerly *Clostridium difficile* [35]) and/or other multidrug-resistant organisms can also result [36]. Women who take ciprofloxacin for cystitis (infection of the urinary bladder, herein abbreviated as UTI) may end up with vaginal yeast overgrowth (typically *Candida albicans*). All of these secondary effects are a consequence of the efficacy of fluoroquinolones against both the infecting bacteria and the commensal flora. The commensal microbiome normally keeps these pathogens in check.

By killing off susceptible microbes and allowing any that are resistant to proliferate, antibiotic treatment also selects for antibiotic resistance. With 10 trillion to 100 trillion bacteria in the human microbiome, the probability that there is one bacterium already present that can resist the antibiotic is high. (This bacterium may have been "born" wicked). Even if this resistant bacterium is not pathogenic, it can share its resistance mechanism with other microbes. Bacteria share DNA with other bacteria through conjugation (transfer of conjugal plasmid from one bacterium to another), transformation (uptake of DNA), or transfection (infection from a bacterial virus, a bacteriophage). Although there has not been an instance reported, and the length of the DNA integrated is short, there is even a chance that the clustered regular interspaced short palindromic repeats (CRISPR) “primitive immune system,” found in nearly half of tested bacterial species (reviewed in [37]), could pass resistance from one microbe to another. Antibiotics stimulate this mutation and genetic exchange among bacteria (reviewed in [38]). In all of these cases, then, wickedness is thrust upon the bacteria. The resulting increase in the prevalence of ciprofloxacin-resistant bacteria thus increases the likelihood of subsequent infection by additional antibiotic-resistant bacteria. Furthermore, because fluoroquinolone resistance is associated with resistance to other classes of antibiotics as well as multidrug resistance [34,39], infection by these microbes would be even more difficult to treat.

As a result of unintended consequences, one of the most common uses of ciprofloxacin, treatment of uncomplicated cystitis (UTI), has changed over the past decade. The 1999 UTI guidelines endorsed by both American and European infectious disease societies recommended treatment with trimethoprim-sulfamethoxazole (Bactrim) or fluoroquinolones but preferred fluoroquinolones in regions where trimethoprim-sulfamethoxazole resistance exceeded 10% to 20% [40]. Partly as a consequence of increasing trimethoprim-sulfamethoxazole resistance and, perhaps, aggressive advertising by Bayer AG during the period of its patent protection, ciprofloxacin gained up to 70% of the UTI treatment market share by 2001 [41]. Just nine years later, the most recent UTI guidelines issued in 2010 no longer recommend the use of fluoroquinolones as a first-line treatment of uncomplicated UTIs because of “collateral damage,” namely selection for increased antibiotic resistance and increased colonization and infection with antibiotic-resistant bacteria and *C*. *difficile* [42].

## One short day

Special attention should be given to *C*. *difficile* infection. Whereas they are an unintended consequence of antibiotic use, they have quickly become an antibiotic-resistant threat. About half of the first symptoms of *C*. *difficile* infection in the United States appear in patients recently or currently hospitalized, with an estimated 250,000 infections and 14,000 deaths per year [43]. Deaths in the US related to *C*. *difficile* increased 400% between 2000 and 2007, partially due to the emergence of a stronger *C*. *difficile* strain [43]. An early 2000s North American epidemic of antibiotic-associated diarrhea was caused by the *C*. *difficile* NAP1/BI/027 strain, which had high fluoroquinolone MICs, increased toxin production, and both more frequent severe infection and greater mortality when compared to nonepidemic *C*. *difficile* strains [44–47]. While the exact events that caused the rapid global emergence of this epidemic strain remain enigmatic, genome comparisons of a collection of epidemic and nonepidemic strains revealed that *C*. *difficile* NAP1/BI/027 consists of two genetically distinct lineages that independently acquired identical mutations in *gyrA* [48]. Thus far, how the *gyrA* mutations affect fluoroquinolone resistance in *C*. *difficile* has not been measured directly. This organism does not have a topoisomerase IV. Genetic alterations, including those affecting drug efflux, likely will be found to combine with these *gyrA* mutations to reach R.

## Thank goodness

To limit the collateral damage and the rise in antibiotic resistance caused by fluoroquinolones, healthcare providers and patients are now encouraged to use this class of antibiotics as responsibly as possible. For treatment of uncomplicated cystitis, nonfluoroquinolone agents, such as nitrofurantoin, fosfomycin (limited availability in the US), and pivmecillinam (not available in the US), are often safe and effective. These antimicrobial agents are considered first-line therapy for UTIs, while fluoroquinolones should be reserved, when possible, for more serious conditions, such as pyelonephritis (kidney infection) or acute diverticulitis (colon infection) [42].

Some people are prescribed long-term antibiotic therapy to prevent recurrent UTIs. This long-term therapy can be effective but carries not only the risk of collateral damage but also side effects from the antibiotics. Although long-term management of recurrent UTIs is challenging, patient health and well-being can instead be achieved through individualized strategies. For motivated patients, one approach is “on-demand” antibiotic therapy, which includes teaching patients to accurately self-diagnose UTIs, even at early symptoms, and immediately begin patient-initiated antibiotic therapy. Postcoital antibiotics are also an option for UTIs associated with sexual activity. Other strategies for recurrent UTIs, such as vaginal microbiome restoration [49], probiotics, cranberry products, and other methods have shown mixed results. Some of these strategies are relatively safe (e.g., cranberry extract) and should not be discouraged, but further investigation is required to establish efficacy.

## Popular

The problem of antibiotic resistance is not exclusive to medical treatment in humans. Antibiotics are used in the agriculture industry for both prophylactic and therapeutic treatment of livestock and are given at lower doses to promote growth and increase feed efficiency. In fact, more antibiotics are used in the agriculture industry than in humans. In 2011, the United States Food and Drug Administration estimated a domestic sale and distribution of 13.6 million kilograms of antimicrobials (61% of which are classified as medically important) for food-producing animals compared with 3.29 million kilograms of antimicrobials approximated for use in humans [50,51]. In 2013, over half of 162,000 tons of antibiotics in China were utilized for livestock breeding [52].

Any antibiotic-resistant bacteria that result in livestock from antibiotic use can potentially cause infections in humans. *Salmonella* and *Campylobacter*, common food-borne bacteria, cause an estimated 410,000 antibiotic-resistant infections in the US each year [43]. In addition, resistant bacteria may also be transferred to humans through contaminated wastewater or fertilizer produced from farming. In the Henan Province of China, for example, 94% of bacteria isolated from fertilizer from three different chicken farms were resistant to at least 5 of 11 tested antibiotics, and nearly half of 61 tested antibiotic resistance genes were present [53].

In the agriculture industry, fluoroquinolone resistance is problematic. Studies in Tunisia and Nigeria have reported high levels of fluoroquinolone resistance in bacteria isolated from cattle (61%–62%) and poultry (42%–55%) [53,54]. The World Health Organization, in compiling their 2017 "List of Critically Important Antibiotics for Human Medicine,” lists quinolones in the top five highest-priority antibiotics, requesting prudent use in human and veterinarian medicine [55].

Whereas antibiotic use for growth promoters was banned by the European Union in 2006, and essentially eliminated through legislation in the US in 2017, relevant legislation does not yet exist in 110 of 130 countries surveyed by the World Organization for Animal Health [56]. It is a challenge to ensure that antibiotics ostensibly reported for medical use in animals are not instead being used as growth promoters. Fortunately, addressing antibiotic resistance is one of three priorities of a tripartite alliance formed by the World Health Organization, the Food and Agriculture Organization of the United Nations, and the World Organization for Animal Health, an encouraging global collaboration in addressing this issue [56]. Any conversation on how to solve the problem of antibiotic resistance, then, must concern itself with use and misuse of antibiotics in the agriculture industry, where the reality of emerging antibiotic resistance must be evaluated relative to economic benefits and the necessities of antibiotic use.

## Defying tragedy

“The thoughtless person playing with penicillin treatment is morally responsible for the death of the [person] who succumbs to infection with the penicillin-resistant organism.”

–Alexander Fleming [57]

Even the discoverer of the first antibiotic knew the global responsibility their use would have to bring. As much as we use antibiotics, such as penicillin or ciprofloxacin, there is still much that we do not know about them. Although bacterial resistance will continue to evolve, we must continue innovating to try to improve treatment options without doing additional harm.

To try and minimize the empirical prescribing problem, investigators are developing rapid molecular diagnostic tests that assay for the presence of certain resistance genes. For such a test to work for fluoroquinolones, however, we would need to know all possible genetic alterations that can cause fluoroquinolone resistance, and we are far from meeting that requirement [33]. Importantly, the mere presence of an antibiotic resistance gene does not necessarily translate to resistance of the bacterium [58–61].

Whereas large-scale genomics and other "-omics" approaches have yielded petabytes of data, much in the datasets are not yet interpreted and none are fully understood. A concerted global systems approach is needed to understand the interactions between host (including host microbiome) and microbes and thus the unknown factors that may cause ciprofloxacin to fail.

As we work to improve our ability to diagnose and treat the super-wicked problem of antibiotic-resistant infections, we must also further understand how the researcher, the clinician, the patient, and the public all affect the continual development of resistance. We must all recognize that antibiotics are a societal good and that the use or misuse by one person can lead to harm for another.
